# Treatment with high-dose n-3 PUFAs has no effect on platelet function, coagulation, metabolic status or inflammation in patients with atherosclerosis and type 2 diabetes

**DOI:** 10.1186/s12933-017-0523-9

**Published:** 2017-04-14

**Authors:** Malgorzata Poreba, Magdalena Mostowik, Aleksander Siniarski, Renata Golebiowska-Wiatrak, Krzysztof Piotr Malinowski, Maciej Haberka, Ewa Konduracka, Jadwiga Nessler, Anetta Undas, Grzegorz Gajos

**Affiliations:** 1 John Paul II Hospital, Pradnicka 80, 31-202 Kraków, Poland; 2 Department of Medical Education, Jagiellonian University Medical College, Kraków, Poland; 3 Institute of Public Health, Faculty of Health Science, Jagiellonian University Medical College, Kraków, Poland; 4 Department of Cardiology, School of Health Science, Medical University of Silesia, Katowice, Poland; 5 Department of Coronary Disease and Heart Failure, Institute of Cardiology, Faculty of Medicine, Jagiellonian University Medical College, Pradnicka 80, 31-202 Kraków, Poland; 6 Institute of Cardiology, Jagiellonian University Medical College, Pradnicka 80, 31-202 Kraków, Poland

**Keywords:** Cardiovascular disease, Atherosclerosis, Diabetes type 2, Omega-3 polyunsaturated fatty acids

## Abstract

**Background:**

Despite numerous studies on cardioprotective effects of omega-3 polyunsaturated fatty acids (n-3 PUFAs), there is limited evidence for n-3 PUFA-mediated effects, especially at its higher dose, on cardiovascular risk in patients with type 2 diabetes (DM2) and established atherosclerosis.

**Purpose:**

To investigate the effect of daily treatment with a higher dose (2 g) of n-3 PUFAs on platelet function, coagulation parameters, fibrin clot properties, markers of systemic inflammation and metabolic status, in patients with atherosclerotic vascular disease and DM2 who receive optimal medical therapy.

**Methods:**

We conducted a prospective, double-blind, placebo-controlled, randomized, double-center study, in which thrombin generation (plasma thrombogenic potential from automated thrombogram), fibrin clot properties (plasma fibrin clot permeability; lysis time), platelet aggregation (light transmission aggregometry with adenosine diphosphate and arachidonic acid used as agonists), HbA1c, insulin level, lipid profiles, leptin and adiponectin levels, as well as markers of systemic inflammation (i.e., hsCRP, IL-6, TNF-α, ICAM-1, VCAM-1, and myeloperoxidase) were determined at baseline and at 3 months after treatment with 2 g/day of n-3 PUFAs (n = 36) or placebo (n = 38). Moreover, we assessed serum fatty acids of the phospholipid fraction by gas chromatography both at baseline and at the end of the study.

**Results:**

Majority of patients were treated with optimal medical therapy and achieved recommended treatment targets. Despite higher serum levels of eicosapentaenoic acid (EPA) (by 204%; p < 0.001) and docosahexaenoic acid (DHA) (by 62%; p < 0.0001) in n-3 PUFA group at the end of treatment no changes in platelet aggregation, thrombin generation, fibrin clot properties or markers of systemic inflammation were observed. No intergroup differences in the insulin, HbA1c and lipid levels were found at the end of the study. There was no change in adiponectin and leptin in interventional group, however leptin increased in control group (p = 0.01), therefore after study period leptin levels were lower in the interventional group (p = 0.01). Additionally, resolvin D1 did not differ between interventional and control group.

**Conclusions:**

In conclusion, our study demonstrated that in patients with long-standing, well-controlled DM2 and atherosclerotic disease the treatment with a high dose of n-3 PUFAs (namely, 1 g/day of EPA and 1 g/day of DHA for 3 months) does not improve coagulation, metabolic, and inflammatory status when measured with the specified tests.

The study was registered in ClinicalTrials.gov; identifier: NCT02178501. Registration date: April 12, 2014

**Electronic supplementary material:**

The online version of this article (doi:10.1186/s12933-017-0523-9) contains supplementary material, which is available to authorized users.

## Background

Numerous studies have provided evidence for pleiotropic and cardioprotective effects of omega-3 polyunsaturated fatty acids (n-3 PUFAs). Currently, with advances in pharmacological and interventional cardiovascular (CV) therapy, the results of major randomized controlled trials (RCTs) have challenged the beneficial effect of n-3 PUFAs on CV risk [1, 2]. On the other hand, a recently published RCT, with a higher dose of n-3 PUFAs, has shown their favorable effects in patients after acute myocardial infarction [3]. However, no RCTs so far have assessed the effects of a higher dose of n-3 PUFAs on CV risk in patients with type 2 diabetes (DM2) and established atherosclerosis.

Diabetes is a well-known prothrombotic and hypofibrinolytic state due to increased platelet reactivity and enhanced secondary hemostasis [4]. Moreover, in patients with DM2 and CV diseases (CVDs), thrombin generation is even more increased than in patients with DM2 without CVDs [5]. Despite advances in modern therapy, patients with diabetes continue to have worse prognosis, and enhanced thrombotic environment has been shown to play a key role in unfavorable clinical outcomes [4]. Moreover, enhanced coagulability has been found to be associated with both microvascular [6] and macrovascular complications [7] in DM2. Although one RCT has suggested no benefits of low-dose n-3 PUFAs in dysglycemia [2], there have been no trials elucidating the mechanisms by which the effectiveness of n-3 PUFAs is reduced in DM2. It is possible that the negative consequences of long-standing, suboptimally controlled DM2, the so called “metabolic memory”, may limit the benefits not only of further intensive glucose control therapy but also of n-3 PUFA supplementation in diabetic patients. The high effectiveness of modern optimal medical therapy (OMT) may also limit the effects of n-3 PUFAs. Moreover, there is limited evidence from RCTs on the impact of n-3 PUFAs on platelets, coagulation, fibrin clot properties, as well as on metabolic balance in high-risk patients with both atherosclerosis and DM2, especially when added to OMT including potent pharmaceuticals [such as statins at high doses and angiotensin-converting-enzyme inhibitors (ACEIs)] and optimal glucose control. The pathogenesis of DM2 is strongly associated with insulin resistance (IR) as one of the major complications of obesity. The adipokine profile deteriorates in obesity and obesity-related conditions, including DM2 [8]. A few adipokines have been suggested to participate in the development of systemic low-grade inflammation, IR and metabolic syndrome [9]. Moreover, disturbances in adipokine profiles have also been associated with inadequate lipid control [10]. Since dysregulation of adipokine secretion plays an important role in the pathogenesis of DM2 and its macrovascular complications, the amelioration of adipokine pattern may improve prognosis in patients with DM2. It has been shown that n-3 PUFAs may improve insulin sensitivity in animal model [11] and modulate adipokine gene expression in animals and humans [12]. However, data supporting the impact of n-3 PUFAs on adipokine profile, systemic inflammation, glucose homeostasis, insulin sensitivity, and lipid metabolism in DM2 are still limited and inconsistent.

The main purpose of the current study was to investigate the effect of daily treatment with a higher dose (2 g) of n-3 PUFAs on platelet function, coagulation parameters, clot properties, as well as markers of systemic inflammation and metabolic status (i.e., HbA1c, insulin level, lipid profiles and leptin and adiponectin levels), in patients with atherosclerotic vascular disease and DM2 who receive OMT.

## Methods

### Patients

**Fig. 1:**
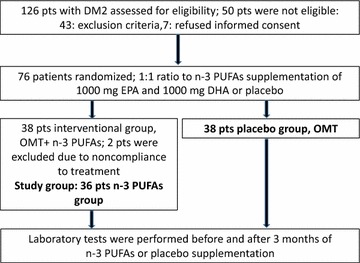
Study flow chart. *n-3 PUFAs* omega-3 polyunsaturated fatty acids, *EPA* eicosapentaenoic acid, *DHA* docosahexaenoic acid, *pts* patients, *OMT* optimal medical therapy

During the period between 04.2014 and 03.2015 we evaluated 126 consecutive patients from the outpatient cardiology department with DM2 and a history of coronary artery disease (CAD) or peripheral artery disease, who fulfilled the study criteria. The exclusion criteria were as follows: pregnancy, type 1 diabetes, poorly controlled DM2 [hemoglobin A1c (HbA1c) >9%], acute coronary syndrome (within the previous 3 months), percutaneous coronary intervention or coronary artery bypass grafting (within the previous month), percutaneous or surgical revascularization (within the previous month), acute infection, hypertriglyceridemia, n-3 PUFA treatment, active bleeding, any known coagulation or bleeding disorder, oral anticoagulant therapy, platelet count <100 × 10^9^/L, serum creatinine levels >177 μmol/L (2 mg/dL), liver injury [alanine transaminase (ALT) levels >1.5 times above the upper limit of the reference range], chronic therapy with nonsteroidal anti-inflammatory drugs, except for acetylsalicylic acid or steroids, alcohol or drug abuse, known sensitivity or allergy to fish or PUFA supplements, history of malignancy (unless disease free for >10 years, or non-melanoma skin carcinoma), life expectancy <12 months due to concomitant diseases, abnormality of laboratory or imaging tests that would hamper the interpretation of results, and any threatening condition during the study (Fig. 1).

### Study design

We conducted a prospective, double-blind, placebo-controlled, randomized, double-center study. In the first month, eligible patients who consented to participate in the study underwent optimization of DM2 and CVD therapy in accordance with the current guidelines. Then, patients were randomized at a ratio of 1:1, using computerized random-number generation by an independent investigator on a double-blind basis to either a n-3 PUFA (OMT plus n-3 PUFA supplementation) or placebo group (OMT). n-3 PUFAs were administered with the Smartfish drink (Smartfish, Oslo, Norway): eicosapentaenoic acid (EPA), 1 g/day, and docosahexaenoic acid (DHA), 1 g/day (stabilized against oxidation by botanical additives: pomegranate, chokeberry, and transresveratrol). The placebo group received the same drink without n-3 PUFAs. Compliance was assessed based on the number of packages of study substance returned at each visit in both groups as well as on plasma concentrations of EPA and DHA. Patients were encouraged to increase their consumption of oily fish and comply with the diet recommended by the European Society of Cardiology. The study complied with the principles of the Good Clinical Practice International Conference on Harmonization rules and was approved by the University Ethics Committee. Each study participant provided written informed consent.

The study was registered in ClinicalTrials.gov; identifier: NCT02178501. Registration date: April 12, 2014.

### Blood sampling and laboratory measurements

Fasting blood samples were obtained between 8 and 10 a.m. after overnight fast. The samples were processed 30–60 min after blood collection and stored at −70 °C until further analysis. Blood for platelet aggregation was not frozen prior to analysis. Blood was taken from the antecubital vein, with minimal stasis at 2 time points: at baseline (i.e., after 1 month of OMT) and after 3 months of n-3 PUFA or placebo supplementation. Routine blood tests, including the measurement of complete blood count, lipid profile, and the levels of aspartate aminotransferase (AST), ALT, and serum creatinine, were done by automated laboratory techniques. HbA1c levels were measured using a turbidimetric inhibition immunoassay.

Platelet aggregation was assessed with the use of light transmission aggregometry. Platelets were stimulated with 10 µmol/L of adenosine diphosphate (ADP) and 5 mmol/L of arachidonic acid. Then, aggregation was measured with the use of a 2-channel Chronolog Aggregometer (Chrono-Log 490; Chrono-Log Corp., Haverton, Pennsylvania) and expressed as the maximum change in light transmission from baseline, in percentages, with platelet-poor plasma as a reference.

Plasma thrombogenic potential was assessed on the basis of a thrombogram, analyzed with the use of the CAT (Thrombinoscope BV, Maastricht, the Netherlands), according to the protocol of the manufacturer in a 96-well plate fluorometer (Ascent Reader, Thermolabsystems OY, Helsinki, Finland) equipped with the 390/460 filter set at 37 °C.

To assess plasma clot lysis, plasmin-mediated fibrinolysis was evaluated in the presence of recombinant tissue plasminogen activator (Boehringer Ingelheim, Ingelheim, Germany). Lysis time was chosen as a marker of the clot susceptibility to fibrinolysis. It was defined as the time needed for a 50% reduction of fibrin clot absorbance.

To assess fibrin clot permeability, calcium chloride (20 mmol/L) and human thrombin (1 U/mL) were added to 120 μL of citrated plasma. After incubation for 120 min, tubes with the clots were connected to a container with a buffer (10 mmol/L: 0.05 mol/L Tris–HCl; 100 mmol/L: 0.15 mol/L NaCl, pH 7.5). Its volume flowing through the gels was measured within 60 min. Then, a permeation coefficient was calculated, indicating the size of fibrin clot pores.

Latex nephelometry was used to measure fibrinogen and high-sensitivity C-reactive protein levels (hsCRP; Dade Behring, Marburg, Germany). The levels of interleukin 6 (IL-6), tumor necrosis factor α (TNF-α), intercellular adhesion molecule 1 (ICAM-1), vascular adhesion molecule 1 (VCAM-1), and myeloperoxidase were measured by an enzyme-linked immunosorbent assay (R&D Systems, USA). An immunoenzymatic assay was used to determine prothrombin fragment 1.2 (F1.2) (Siemens, Marburg, Germany). Plasma prothrombin levels were determined by 1-stage clotting assays using factor-deficient plasma (Dade Behring, Liederbach, Germany). Resolvin D1 (RvD1) levels were measured by the RvD1 EIA Kit (Cayman Chemical Company, USA).

Serum leptin and adiponectin levels were determined by radioimmunoassay kits (DIAsource, Belgium). Insulin levels were measured using the chemiluminescent immunoassay method (Advia Centaur, Siemens Healthcare, USA) with intra- and interassay variabilities ranging from 3.2 to 4.6% and from 2.6 to 5.9%, respectively. The measurement of serum peptide C levels was performed using radioimmunoassay kits (DIAsource, Belgium).

The assessment of body composition was performed using a bioelectrical impedance analysis.

### n-3 PUFA measurement

The analytical procedure consisted of a few separate steps: (1) extraction of serum total lipids; (2) separation of the lipid fraction on Sep-PakNH2 columns (Waters, Milford, Mass., USA); (3) methylation and separation of the fatty acid from the phospholipid fraction by gas chromatography (Agilent Technologies 6890N Network GC Systems, Wilmington, De., USA) equipped with an Agilent J&W HP-88 capillary column (100 m, 0.250 mm, 0.20 µm) (Agilent Technologies, USA). 1,2-dipentadecanoil-sn-glicero-3-phosphocholine (Sigma-Aldrich, Steinheim, Germany) was used as an internal standard. The method was calibrated using the calibration mixture (all fatty acids: Sigma-Aldrich, Steinheim, Germany). The plasma levels of saturated acids (lauric, C12; myristic, C14; palmitic, C16; stearic, C18; lignoceric, C24) and unsaturated acids (n-7 [palmitoleic, C16:1], n-9 [oleic, C18:1], n-3 [alfa-linolenic, C18:3; EPA, C20:5; DHA, C22:6], and n-6 [linoleic, C18:2; eicosadienoic C20:2; arachidonic -AA, C20:4] of the phospholipid fraction were quantitatively measured by gas chromatography. The concentration of serum fatty acids of the phospholipid fraction was expressed in µmol/L.

All laboratory tests were performed by investigators blinded to the sample origin.

### Statistical analysis

Categorical variables were presented as numbers and percentages. Continuous variables were expressed as mean ± standard deviation (SD) or median and interquartile range (IQR). Differences between the groups were compared using the Student’s or the Welch’s t test depending on the equality of variances for normally distributed variables. The Mann–Whitney U test was used for nonnormally distributed continuous variables. Normality was assessed by the Shapiro–Wilk test. The equality of variances was assessed using the Levene’s test. Categorical variables were compared by the Fisher’s exact test for 2 × 2 tables or by the Pearson’s Chi squared test for other tables. For pairwise comparisons, the paired t test was used if the difference between variables was normally distributed. The Wilcoxon signed-rank test was used for nonnormally distributed differences. The Pearson correlation coefficient was computed to measure the linear dependence between two normally distributed variables. The Spearman’s rank correlation coefficient was calculated to measure monotonic trend between two variables if the distribution of the variables was nonnormal. Two-sided p values <0.05 were considered statistically significant. All calculations were done with JMP^®^, Version 9.0.0 SAS Institute Inc., Cary, NC, 1989–2007.

The sample size was calculated based on our previous studies [13, 14]. The primary endpoint of this study was the change in the levels of markers of thrombin generation, fibrin clot properties, and platelet aggregation after 3 months of treatment with n-3 PUFAs. The secondary endpoint was the improvement in the markers of systemic inflammation and metabolic status. We hypothesized that the addition of n-3 PUFAs to OMT compared with placebo would result in an absolute 14% reduction of the maximum thrombin concentration with an SD of the differences between the two groups of 13%. We speculated that the addition of n-3 PUFAs to OMT compared with placebo would result in an absolute 15% increase of permeation coefficient with an SD of the differences between the two groups of 15%. We also hypothesized that the addition of n-3 PUFAs to OMT compared with placebo would result in an absolute 13% reduction of the maximum platelet aggregation induced by 5 µmol/L of ADP with an SD of the differences between the two groups of 13%. Choosing a power of 90% and a 2-sided alpha level of 0.05, at least 23 patients in each group were required.

## Results

### Baseline characteristics

A total of 74 consecutive patients (49 men and 25 women; mean age, 65.6 ± 6.8 years) were analyzed (Fig. 1). Baseline demographic data, clinical characteristics, laboratory results, and the use of concomitant medications in randomized patients are shown in Table 1. There were no differences at baseline between the groups, except for lower adiponectin (p = 0.01) and higher triglyceride (TG) levels (p = 0.01) in the n-3 PUFA group (Tables 1, 2, 3; Additional file 1: Table S1).

**Table 1:** Baseline characteristics of the study population (n = 74)

Variables	Placebo N = 38	n-3 PUFAs N = 36
Age (years)	66.7 (±6.8)	64.4 (±6.7)
Female sex, n (%)	12 (31.6)	14 (38.9)
Hypertension, n (%)	36 (93.7)	36 (100)
Hyperlipidemia, n (%)	22 (57.9)	28 (77.8)
Metabolic syndrome, n (%)	38 (100)	36 (100)
Obesity, n (%)	25 (65.8)	23 (63.9)
Waist circumference (cm)	107 (98; 113)	106 (99; 112)
Body mass index (kg/m^2^)	31.1 (28.1; 32.7)	30.9 (27.9; 34.7)
Body fat (%)	30.4 (27.4; 35.8)	34.0 (31.0; 43.0)
Visceral fat (%)	15.74 (±4.9)	16.25 (±4.5)
Total body water (%)	47.9 (43.9; 49.8)	47.5 (44.2; 49.2)
Muscle mass (kg)	53.5 (±11.5)	56.85 (±9.2)
DM2 duration (years)	10 (6; 15)	10 (6.5; 15)
CAD, n (%)	38 (100)	36 (100)
PAD, n (%)	14 (38.9)	12 (31.3)
Previous MI, n (%)	16 (42.1)	12 (33.3)
Previous PCI, n (%)	25 (65.8)	22 (61.1)
Creatinine (μmol/L)	84 (66; 94)	86.5 (74; 100)
eGFR (MDRD) (mL/min/1.73 m^2^)	78.6 (70; 90)	78 (69; 89)
Treatment, n (%)		
Beta blocker	31 (81.6)	30 (83.3)
ACEI or ARB	33 (86.8)	34 (94.4)
Long acting nitrate	5 (13.2)	6 (16.7)
Calcium antagonist	13 (34.2)	19 (52.8)
Statin	33 (86.8)	35 (97.2)
Fibrate	0	1 (2.78)
ASA	38 (100)	36 (100)
Clopidogrel	19 (50)	14 (38.9)
Metformin	24 (63.2)	25 (69.4)
Sulfonylurea	14 (36.8)	17 (47.2)
Acarbose	1 (2.6)	2 (5.6)
DPP-IV	1 (2.6)	0
Insulin	16 (42.1)	16 (44.4)
PPI	10 (26.3)	12 (33.3)
Loop diuretic	6 (15.8)	3 (8.3)
Diuretic	11 (28.9)	11 (30.6)
AA	6 (15.8)	3 (8.3)

Data shown as number (percentage) for categorical variables and mean (±standard deviation) or median (IQR) for continuous variables

*CAD* coronary artery disease, *PAD* peripheral artery disease, *MI* myocardial infarction, *PCI* percutaneous coronary intervention, *eGFR (MDRD)* estimated glomerular filtration rate calculated by the abbreviated MDRD equation, *ACEI* angiotensin-converting enzyme inhibitor, *ARB* angiotensin II receptor blocker, *ASA* acetylsalicylic acid, *PPI* proton pump inhibitors, *AA* aldosterone antagonist

**Table 2:** Effects of 3 months of 2-g n-3 PUFA supplementation on clot properties, coagulation parameters and platelet function

Variable	Placebo	n-3 PUFAs	p value
Fibrinogen (g/L)			
Before	2.70 (2.60; 2.95)	2.74 (2.60; 2.97)	0.70
After	2.73 (2.58; 2.85)	2.72 (2.57; 2.87)	0.91
t50% (min)			
Before	13.14 (±2.77)	13.55 (±3.07)	0.55
After	12.58 (±2.70)	13.76 (±2.59)	0.07
Lag time (min)			
Before	2.94 (2.52; 3.27)	2.61 (2.33; 3.21)	0.35
After	2.94 (2.60; 3.28)	2.84 (2.56; 3.17)	0.75
Peak (nM)			
Before	264.54 (220.08; 314.92)	247.05 (211.89; 287.34)	0.27
After	249.59 (190.94; 336.52)	243.83 (207.82; 293.65)	0.98
tt peak (min)			
Before	5.87 (±0.92)	5.94 (±1.03)	0.76
After	5.62 (5.00; 6.96)	5.98 (4.96; 6.89)	0.89
F1.2 (pg/mL)			
Before	41.40 (28.80;48.10)	34.70 (26.95; 46.85)	0.56
After	43.70 (33.90; 49.30)*	37.70 (27.40; 41.55)**	0.04
PR ADP (%)			
Before	57.96 (±14.02)	54.44 (±14.27)	0.36
After	60.56 (±15.12)	58.19 (±12.52)	0.54

Data shown as number (percentage) for categorical variables and mean (±standard deviation) or median (IQR) for continuous variables

*Ks* permeation coefficient, *t50%* clot lysis time, *Lag time* lag time peak height, *ETP* endogenous thrombin potential (an area under the thrombin concentration versus the time curve), *Peak* maximal thrombin concentration, *tt peak* time do peak, *F1.2* prothrombin fragments, *PR ADP* platelet aggregation 10 µmol/L adenosine diphosphate, *PR AA* platelet aggregation 5 mmol/L arachidonic acid

* p = 0.05 within the placebo group; ** p = 0.53 within the n-3 PUFA group

**Table 3:** Effects of 3 months of 2-g n-3 PUFAs supplementation on metabolic status, inflammatory markers and resolvin D1

Variables	Placebo N = 38	n-3 PUFAs N = 36	p value
HbA1c (%)			
Before	7.00 (6.50; 7.50)	7.00 (6.70; 7.48)	0.45
After	6.80 (6.50; 7.35)	7.10 (6.90; 7.75)	0.06
Insulin (mU/mL)			
Before	22.30 (16.20; 33.63)	20.85 (12.85; 34.80)	0.59
After	16.80 (12.83; 32.23)	22.35 (13.33; 39.58)	0.72
Peptide C (pmol/mL)			
Before	2.99 (2.10; 4.07)	3.27 (2.70; 4.11)	0.43
After	2.87 (1.97; 4.03)	3.30 (2.41; 4.64)	0.15
TC (mmol/L)			
Before	3.53 (3.16; 4.21)	3.80 (3.25; 4.63)	0.50
After	3.78 (3.25; 4.45)	3.50 (3.11; 4.35)	0.53
LDL-C (mmol/L)			
Before	1.84 (1.47; 2.48)	2.07 (1.62; 2.83)	0.51
After	2.07 (1.71; 2.54)	1.81 (1.44; 2.59)	0.24
HDL-C (mmol/L)			
Before	1.23 (1.04; 1.56)	1.22 (0.90; 1.39)	0.45
After	1.24 (0.99; 1.47)	1.19 (0.91; 1.56)	0.76
TG (mmol/L)			
Before	1.30 (0.95; 1.62)	1.79 (1.8; 2.41)	0.01
After	1.41 (1.13; 1.72)	1.48 (0.91; 2.08)	0.69
Leptin (ng/mL)			
Before	5.66 (2.54; 9.14)	3.26 (1.21; 8.09)	0.09
After	6.14 (3.02; 12.19)*	3.45 (1.35; 8.18)	0.01
Adiponectin (ng/mL)			
Before	4.03 (3.21; 6.35)	3.52 (2.32; 4.06)	0.01
After	4.19 (3.20; 5.75)	3.26 (2.28;4.42)	0.03
hsCRP (mg/L)			
Before	1.71 (0.72; 3.10)	1.52 (0.76; 2.53)	0.73
After	1.97 (0.98; 3.13)	1.75 (0.66; 3.73)	0.67
IL-6 (pg/mL)			
Before	2.03 (1.49; 3.32)	1.97 (1.57;2.38)	0.66
After	1.95 (1.19; 3.76)	2.05 (1.69; 3.43)	0.50
TNF-α (pg/mL)			
Before	1.48 (1.29; 1.72)	1.51 (1.26; 1.84)	0.66
After	1.47 (1.25; 1.86)	1.46 (1.21; 1.76)	0.73
sICAM-1/CD54 (ng/mL)			
Before	205.87 (169.4;258.7)	222.64 (168.2; 241.2)	0.94
After	201.88 (168.0; 262.4)	208.27 (178.3; 271.6)	0.74
sVCAM-1/CD106 (ng/mL)			
Before	875.20 (659.8; 1385.0)	1138.2 (818.6; 1604.3)	0.11
After	832.95 (638.3; 1515.0)	1275.3 (825.9; 1554.9)	0.06
Myeloperoxidase (ng/mL)			
Before	28.80 (18.43; 40.68)	40.46 (20.16; 55.57)	0.15
After	28.24 (18.56; 44.92)	29.18 (24.21; 48.99)	0.60
RvD1 (pg/mL)			
Before	244.60 (150.1; 457.5)	237.40 (123.40; 469.0)	0.59
After	232.85 (144.65; 409.4)	184.90 (106.08; 660.6)	0.74

Data shown as number (percentage) for categorical variables and mean (±standard deviation) or median (IQR) for continuous variables

*HbA1c* glycated hemoglobin, *TC* total cholesterol, *LDL-C* low-density lipoprotein cholesterol, *HDL-C* high-density lipoprotein cholesterol, *TG* triglycerides, *hsCRP* high-sensitivity C-reactive protein, *IL-6* interleukin-6, *TNF-α* tumor necrosis factor-α, *ICAM-1* intercellular adhesion molecule-1, *VCAM-1* vascular adhesion molecule-1, *RvD1* resolvin D1

* p = 0.01 within the placebo group

### Coagulation parameters

**Fig. 2:**
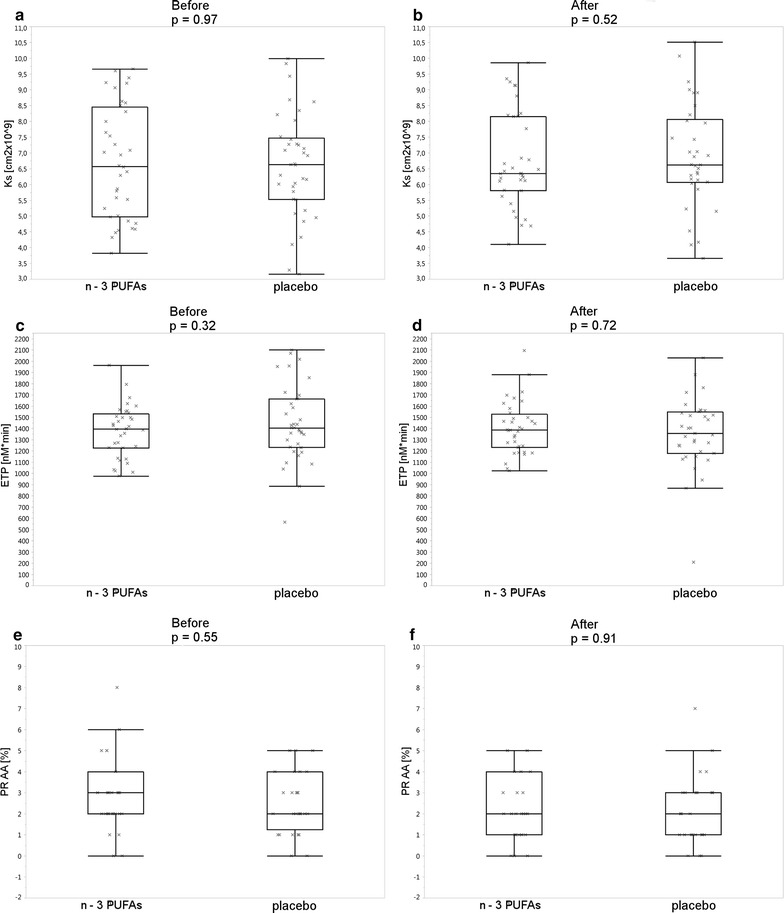
Permeation coefficient (Ks), before (**a**) and after intervention (**b**); endogenous thrombin potential (ETP), before (**c**) and after intervention (**d**); platelet aggregation—5 mmol/L of arachidonic acid (PR AA) before (**e**) and after intervention (**f**) in placebo and n-3 PUFAs group. *Horizontal line* median; *upper and lower margin of rectangle* interquartile range, *vertical line* observation away from 1.5 quartiles

At baseline there were no significant intergroup differences in the markers of thrombin generation and fibrin clot properties. After 3 months of n-3 PUFA treatment, no intergroup differences were found in fibrin clot properties and most markers of thrombin generation, except for F1.2. A within-group analysis showed that there was a trend towards an increase in F1.2 levels in the placebo group (p = 0.05) during the study period, while this parameter remained unaltered in the n-3 PUFA group (p = 0.53). As a result, F1.2 levels were lower in the n-3 PUFA group (p = 0.04) at the end of the study, but the differences were of borderline significance (Table 2; Fig. 2).

### Platelet function

Platelet aggregation measured by specified test described above, did not differ between the groups at baseline and did not change after 3 months of treatment, with no intergroup posttreatment differences (Table 2; Fig. 2).

### Glucose and metabolic parameters

During the n-3 PUFA treatment, no significant changes in the levels of HbA1c, insulin, or peptide C were observed in either interventional or placebo group; therefore, no intergroup differences were found at the end of the study (Table 3).

No significant intergroup differences in the levels of TG, total cholesterol (TC), low-density lipoprotein cholesterol (LDL-C), and high-density lipoprotein cholesterol (HDL-C) were found after the n-3 PUFA treatment. However, there was a trend towards a decrease of TG levels in the interventional group (p = 0.125) and a trend towards an increase of TG levels in the placebo group (p = 0.09) (Table 3).

Both, before and after the treatment with n-3 PUFAs, adiponectin levels were higher in the interventional group than in placebo group (p = 0.01 and p = 0.03, respectively). There was no change in adiponectin levels during the study period in either of the groups (Table 3).

At baseline, there was a trend towards higher leptin levels in the placebo group (p = 0.09). Moreover, leptin levels increased in this group during the study period (p = 0.01). Therefore, after 3 months of treatment with n-3 PUFAs, leptin levels were lower in the interventional group (p = 0.01) (Table 3).

### Markers of inflammation and resolvin D1

After 3 months of treatment with n-3 PUFAs, the markers of inflammation (hsCRP, IL-6, TNF-α, ICAM-1, VCAM-1, and myeloperoxidase) did not differ between the interventional and placebo groups. Despite an increase of both EPA and DHA levels in the n-3 PUFA group, rvD1 levels did not differ between the groups (Table 3).

### Fatty acids

After 3 months of treatment with n-3 PUFAs, DHA levels increased by 154.5 µmol/L (54.5%) in the interventional group and were higher by 61.6% in the n-3 PUFA group compared with placebo (p < 0.0001). EPA levels increased by 101.5 µmol/L (161.2%) in the n-3 PUFA group and were higher by 204.3% in the interventional group compared with the placebo group (p < 0.0001). At the end of the study, the n-6:n-3 ratio was lower by 57.3% in the n-3 PUFA group compared with placebo (p < 0.0001) (Additional file 1: Table S1, Additional file 2: Figure S1).

### Adverse events

The observed adverse events were rated as mild and did not require medical intervention. Their frequency was similar in both groups. Two patients (from both groups each) withdrew from the study during the run-in period because of noncompliance to the study protocol due to diarrhea (n = 1) and nausea (n = 1). There were no major cardiac adverse events (MACEs) during the follow-up in either of the groups.

## Discussion

Our study demonstrated that a 3-month treatment with a higher dose of n-3 PUFAs (1 g of EPA and 1 g of DHA acids), added to OMT in patients with CVD and with long-standing and well-controlled DM2 did not affect platelets aggregation and coagulation, when measured by specified tests, which were used. Moreover, it did not influence inflammatory or metabolic status. This is the first study that attempted to elucidate, in a comprehensive manner, the various mechanisms responsible for the effect (or lack of it) of treatment with n-3 PUFAs in patients with atherosclerosis and DM2. The ORIGIN (Outcome Reduction With Initial Glargine Intervention) study demonstrated no effect of a lower dose of PUFAs (1 g daily) on HbA1c levels or MACE and all-cause mortality in patients with DM2 and prediabetes [2]. However, recently, Kwong et al. [3] have shown that 4 g of PUFAs daily were associated with reduction of adverse left ventricular remodelling, non-infarct myocardial fibrosis, and serum biomarkers of systemic inflammation, in patients with acute myocardial infarction, on guidelines-based therapies. The discrepancies in the effectiveness of treatment with a higher dose of n-3 PUFAs between the studies may be explained by the differences in study populations and protocols. In the study by Kwong et al. [3], acute myocardial infarction was present in about 45% of the patients, while in our study, stable CAD and/or peripheral artery disease and DM2 was diagnosed in all patients. The procoagulant state related to DM2 may be so potent that it cannot be attenuate or abolish by the subtle effects of n-3 PUFAs, which most likely are present in diabetic patients. Moreover, the higher dosage of n-3 PUFAs, 4 g for 6 months used by Kwong et al. [3] versus 2 g for 3 months in our study, might have affected the results. In our study, no significant differences were found in platelet aggregation and fibrin clot properties between the n-3 PUFA and placebo groups during the 3 months of treatment. Although, we noticed a significant intergroup difference in F1.2 levels at the end of the study. This marker of in vivo thrombin generation might suggest that in the placebo group thrombin formation is more prone to enhancement with time, while the therapy attenuates such effect. It remains to be established whether this phenomenon is mediated by increased TF expression in high risk CV patients. However, we cannot exclude that this was an artifact probably caused by a limited study population.

The beneficial effect of n-3 PUFAs on the blood coagulation has been observed in health and disease states in numerous small-scale clinical studies [15]. Supplementation of 1 g/day of n-3 PUFAs for 1 month has been shown to exert antithrombotic effects and potentiate platelet response to clopidogrel in patients with stable CAD, who underwent percutaneous coronary intervention [13, 16]. One month of 1 g of n-3 PUFAs daily has also been found to alleviate the hypercoagulable state by decreasing thrombin formation and improving plasma fibrin clot properties in those patients [14]. Such effects were not observed in patients with DM2 as reported in the present study.

Data concerning the effects of n-3 PUFAs on the coagulation profile in diabetic patients are scarce and inconsistent. In contrast to our results, a reduction in platelet aggregation was observed after 8 weeks of treatment with either 1800 or 900 mg of EPA daily in patients with noninsulin-dependent diabetes without CVD [17]. In another study, 3 g of n-3 PUFA given for 8 weeks in noninsulin-dependent diabetes increased plasminogen activator inhibitor-1 levels, resulting in the impairment of fibrinolytic capacity [18]. However, the limitations of the above study was a small study population (14 patients) and the use of placebo made from olive oil, which is a rich source of biologically active monounsaturated fatty acids.

Discrepant results of the studies might also be explained by the fact that in the current study, we placed a special emphasis on optimization of therapy and most of our participants underwent intensification of lipid control, mainly by increasing statin doses, before enrollment. It has been shown that statins improve a procoagulant status in DM2 [19]. In our study, the mean doses of atorvastatin, rosuvastatin, and simvastatin were 30.5, 16.5 and 22.9 mg, respectively. Thus, it is possible that the anticoagulant properties of statins might have overwhelmed the effects of n-3 PUFAs. Moreover, high fasting TG levels were shown to predict the potential of n-3 PUFAs to decrease the levels of several coagulation factors (V, VII, and X) and reduce thrombin generation [20]. Since the mean TG concentration in our patients was low (1.35 mmol/L) owing to intensive lipid control, it might have also limited the effect of n-3 PUFAs on the coagulation status.

Our results suggest that the higher dose of n-3 PUFAs in optimally treated patients with DM2 and CVD does not improve the metabolic status, including lipid control, insulin sensitivity, and adipokine profile. However, we may hypothesize that due to a high degree of metabolic dysregulation in obesity and DM2, a 3-month treatment with 2 g of n-3 PUFAs might not be potent enough to improve the endocrine activity of fat tissue. Moreover, the high effectiveness of modern OMT may limit the beneficial effects of n-3 PUFAs. Therefore, it is possible that higher doses and longer therapy with n-3 PUFAs would have improved the metabolic status in DM2. On the other hand, due to “metabolic memory” observed in DM2, suboptimal glucose control in the early stages of DM2, especially long-term disturbances in the glucose profile, might explain the lack of metabolic benefits of n-3 PUFA supplementation, even after the intensification of treatment.

The available RCTs investigating the effect of n-3 PUFAs on glucose metabolism showed inconsistent findings, demonstrating mainly neutral or slightly beneficial effects. Several studies suggested the usefulness of n-3 PUFAs in DM2 prevention [21–23]. Nevertheless, the effect of n-3 PUFAs in those studies might have been stronger due to the lack of DM2 and lower degree of metabolic disturbances as compared to our study.

A recent study by Sawada et al. [24] in patients with newly-diagnosed impaired glucose metabolism, including both diabetes and impaired glucose tolerance (IGT), accompanied by CAD, has shown favorable effects of 1800 mg of EPA daily on postprandial hyperglycemia, hyperlipidemia, and insulin secretion. However, no changes were noticed in fasting immune reactive insulin or HbA1c levels. Compared to our study, it was an open-label, single-blind study, where only EPA without DHA was supplemented. There were also differences in study population: patients with CAD and newly diagnosed diabetes or IGT in the study by Sawada et al. [24] versus patients with CVD and long-standing DM2 in our study. Thus, higher impairment of the metabolic status in our study population may be assumed. Sarboluki et al. [25] demonstrated that a 3-month treatment with 2 g of EPA in overweight Asian patients with DM2 improved glucose control indices such as insulin, fasting plasma glucose (FPG), HbA1c, and homeostasis model assessment of insulin resistance (HOMA-IR). However, compared to our study, their population was younger, with a higher percentage of women, lower mean body mass index, shorter duration of DM2, and without insulin treatment. On the other hand, a recent meta-analysis by Chen et al. [26] demonstrated that in DM2 n-3 PUFAs did not change HbA1c, fasting insulin, FPG, or postprandial glucose levels, which is in accordance with our results. Nonetheless, most RCTs included in the meta-analysis were carried out within a period of 3 months, and it may be speculated that longer supplementation might have improved glycemic control.

Also a few trials evaluating higher doses of n-3 PUFAs (4 g/day of n-3 PUFAs and 3–18 g/day of fish oil) than the dose used in our study (2 g) supported our findings of no effect of n-3 PUFAs on glucose metabolism in DM2 [27, 28].

Regarding n-3 PUFA-mediated effects on adiponectin and leptin levels in patients with DM2, our observations are in agreement with the studies by Striban et al. [29], where 2 g daily of EPA/DHA were administered, and Kabir et al. [30], where patients received 1.8 g/day of n-3 PUFAs. A recent meta-analysis demonstrated that n-3 PUFA therapy is mildly associated with decreased plasma leptin levels only in nonobese participants [31], which is in line with our results. Although n-3 PUFAs might moderately increase adiponectin levels [32], the only study performed in patients with DM2 demonstrated no impact of n-3 PUFAs on adiponectin levels.

In the present study, after treatment with a high dose of n-3 PUFAs, EPA and DHA concentrations were very high with no change in RvD1 levels. During the study, n-3 PUFAs exerted no significant effect on inflammatory markers such as hsCRP, IL-6, TNF-α, ICAM-1, VCAM-1, or myeloperoxidase. We hypothesize that the lack of the anti-inflammatory properties of n-3 PUFAs in DM2 may result from impaired synthesis of their active metabolites associated with long-term advanced DM2 and atherosclerosis.

Our study has several limitations. First, the duration of treatment was relatively short and the sample size was limited. However, our previous studies showed that the benefits of n-3 PUFA supplementation can be seen in a 1-month observation. Second, we did not assess clinical endpoint. A certain impact of potential confounders such as diet, physical activity, or cigarette smoking cannot be excluded.

## Conclusions

In conclusion, our study demonstrated that in patients with long-standing, well-controlled DM2 and atherosclerotic disease the treatment with a high dose of n-3 PUFAs (namely, 1 g/day of EPA and 1 g/day of DHA for 3 months) does not improve coagulation, metabolic, and inflammatory status when measured with the specified tests. Further studies are needed to clarify a role of n-3 PUFAs in the regulation of complex processes active in vivo.

## Additional files

**Additional file 1: Table S1.** Concentration of serum fatty acids of the phospholipid fraction before and after 3 months of either placebo or n-3 PUFA treatment.

**Additional file 2: Figure S1.** Docosahexaenoic acid; C22:6 (DHA), before A and after intervention B; Resolvin D1 (RvD1), before C and after intervention D; n-6: n-3 ratio before E and after intervention F in placebo and n-3 PUFAs group. The n-6:n-3 ratio was calculated by measuring C18:2n-6; C20:2n-6; C204n:-6 acids to estimate total n-6 fatty acids and C18:3n-3, C20:5n-3 and C22:6n-3 to estimate total n-3 fatty acids. Horizontal line—median; upper and lower margin of rectangle—interquartile range, vertical line—observation away from 1.5 quartiles. Abbreviation as Additional file 1: Table S1.
