# Supplementary material for: Treatment with high-dose n-3 PUFAs has no effect on platelet function, coagulation, metabolic status or inflammation in patients with atherosclerosis and type 2 diabetes
Source: Cardiovasc Diabetol. 2017 Apr 14;16:50. doi: 10.1186/s12933-017-0523-9 (PMC5391604; doi:10.1186/s12933-017-0523-9)
Supplement: Supplementary file 1 — Additional file 1: Table S1. Concentration of serum fatty acids of the phospholipid fraction before and after 3 months of either placebo or n-3 PUFA treatment. [file 12933_2017_523_MOESM1_ESM.docx]

**Supplementary Material**

**Table S1. Concentration of serum fatty acids of the phospholipid fraction before and after 3 months of either placebo or n-3 PUFA treatment.**

| **Variables** | **Placebo**  **N=38** | **n-3 PUFAs**  **N=36** | **p-value** |
| --- | --- | --- | --- |
| C12 (µmol/l)  Before  After | 2.08 (1.31; 3.14)  1.83 (1.43; 2.68) | 1.76 (1.30; 3.32)  1.85 (1.29; 3.60) | 0.89  0.795 |
| C14 (µmol/l)  Before  After | 16.40 (14.04; 19.89)  17.06 (14.29; 20.14) | 17.33 (14.83; 19.23)  15.11 (13.67; 20.27) | 0.71  0.45 |
| C16 (µmol/l)  Before  After | 946.31(828.34; 1079.57)  975.42 (861.97; 1110.27) | 955.95 (881.07; 1072.16  986.27 (827.33; 1204.30) | 0.50  0.91 |
| C18 (µmol/l)  Before  After | 450.32 (336.30; 513.79)  484.23 (393.22; 530.75) | 402.38 (365.36; 463.72)  396.15 (342.41; 531.54) | 0.665  0.115 |
| C24 (µmol/l)  Before  After | 24.70 (21.81; 27.35)  23.05 (19.99; 29.70) | 23.48 (17.96; 30.69)  22.68 (18.32; 28.20) | 0.68  0.47 |
| C16:1 (µmol/l)  Before  After | 14.76 (10.34; 21.86)  16.65 (10.75; 22.52) | 15.09 (13.80; 18.86)  15.55 (11.27; 20.35) | 0.34  0.83 |
| C18:1 (µmol/l)  Before  After | 288.13 (±69.47)  304.42 (±72.14) | 299.30 (±76.66)  279.51 (±71.65) | 0.52  0.14 |
| C18:3 (µmol/l)  Before  After | 6.30 (4.85; 9.72)  6.84 (5.43; 9.04) | 6.45 (4.78; 8.69)  5.64 (4.09; 6.99) | 0.73  0.07 |
| C20:5; EPA (µmol/l)  Before  After | 60.73 (42.67; 80.92)  54.04 (36.73; 73.82) | 62.94 (45.17; 82.51)  164.42 (132.51; 203.77)** | 0.72  <0.0001 |
| C22:6; DHA (µmol/l)  Before  After | 289.57 (242.59; 327.43)  270.97 (217.79;362.07) | 283.51 (230.39; 343.82)  437.91 (360.32; 512.80)** | 0.66  <0.0001 |
| C18:2(µmol/l)  Before  After | 439.23 (378.39; 538.20)  465.48 (370.30; 525.92) | 438.84 (358.21; 529.38)  378.29 (320.11; 485.52)* | 0.965  0.03 |
| C20:4; AA (µmol/l)  Before  After | 543.66 (±100.48)  573.14 (±113.03) | 560.06 (±151.06)  437.32 (±108.30)** | 0.59  <0.0001 |
| C20:2 (µmol/l)  Before  After | 17.10 (13.38;21.30)  18.39 (15.43;23.09) | 16.29 (14.78;20.77)  14.24 (12.05;20.49) | 0.85  0.01 |
| n-6 : n-3 ^1^  ratio  Before  After | 2.8(2.37; 3.44)  3.09(2,63; 634) | 3.11(2,25; 3.78)  1.32(1.13; 1.68)** | 0.42  < 0.0001 |

Data shown as number (percentage) for categorical variables and mean (±standard deviation) or median (IQR) for continuous variables.

^1^ **The n-6: n-3** **ratio** was calculated by measuring: linoleic, C18:2n-6; eicosadienoic, C20:2n-6; and arachidonic -AA, C20n:4-6 acids to estimate total n-6 fatty acids and C18:3n-3, alfa-linolenic; C20:5n-3; C22:6n-3 to estimate total n-3 fatty acids; * p=0.003 within the n-3 PUFA group, ** p < 0.0001 within the n-3 PUFAs group. Abbreviations as in the text of manuscript.
