# Supplementary figures and images for: Treatment with high-dose n-3 PUFAs has no effect on platelet function, coagulation, metabolic status or inflammation in patients with atherosclerosis and type 2 diabetes
Source: Cardiovasc Diabetol. 2017 Apr 14;16:50. doi: 10.1186/s12933-017-0523-9 (PMC5391604; doi:10.1186/s12933-017-0523-9)

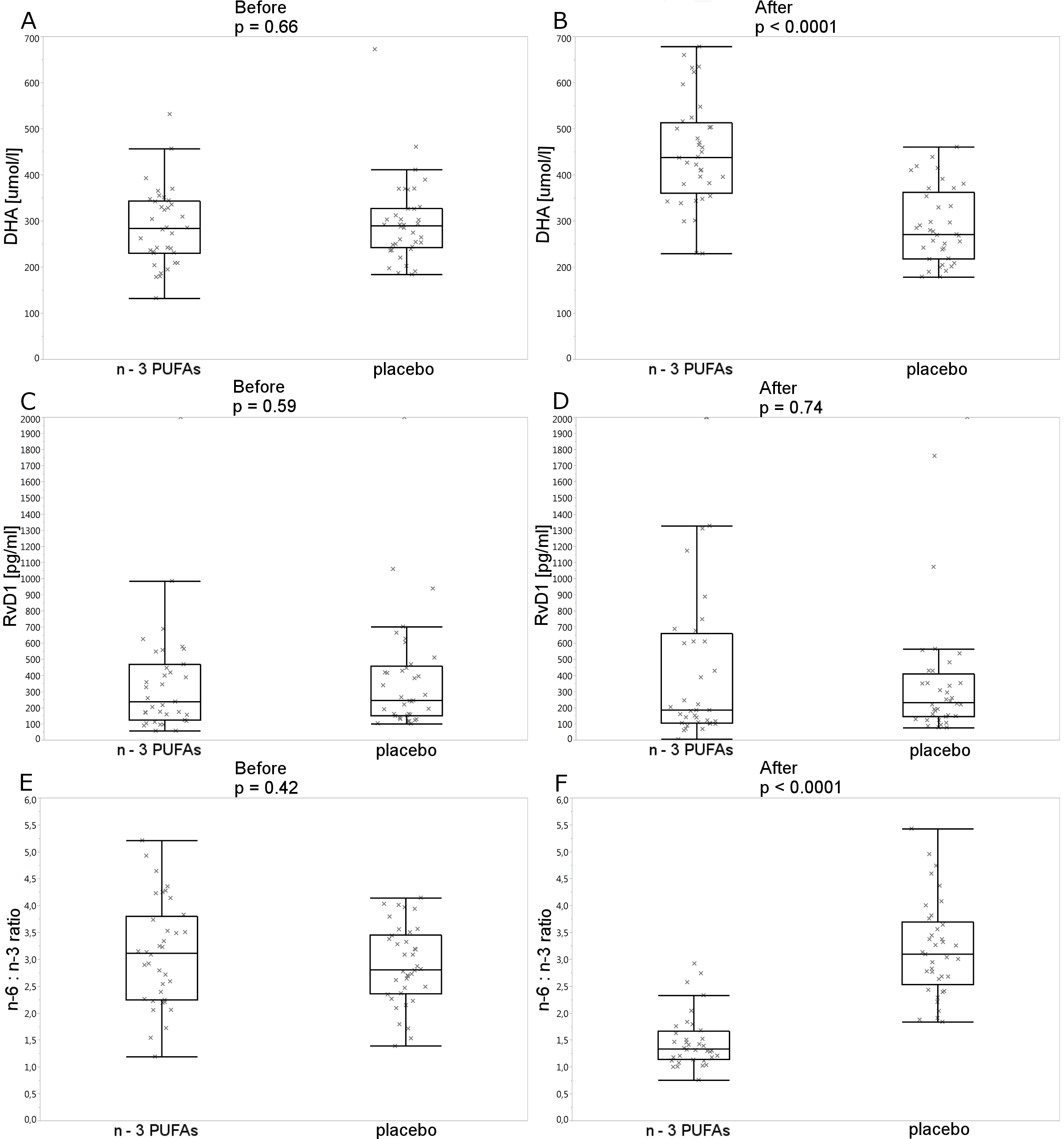

Supplement: Supplementary file 2 — Additional file 2: Figure S1. Docosahexaenoic acid; C22:6 (DHA), before A and after intervention B; Resolvin D1 (RvD1), before C and after intervention D; n-6: n-3 ratio before E and after intervention F in placebo and n-3 PUFAs group. The n-6:n-3 ratio was calculated by measuring C18:2n-6; C20:2n-6; C204n:-6 acids to estimate total n-6 fatty acids and C18:3n-3, C20:5n-3 and C22:6n-3 to estimate total n-3 fatty acids. Horizontal line—median; upper and lower margin of rectangle—interquartile range, vertical line—observation away from 1.5 quartiles. Abbreviation as Additional file 1: Table S1. [file 12933_2017_523_MOESM2_ESM.tiff]
